# Contemporary small-scale subsistence populations offer unique insights into human musculoskeletal health and aging

**DOI:** 10.1126/sciadv.adq1039

**Published:** 2024-11-08

**Authors:** Jonathan Stieglitz

**Affiliations:** Department of Social and Behavioral Sciences, Toulouse School of Economics, Institute for Advanced Study in Toulouse, Université Toulouse Capitole, Toulouse, France.

## Abstract

Human foragers avoid noncommunicable diseases that are leading causes of mortality, partly because physically active lifestyles promote healthy aging. High activity levels also promote tissue damage accumulation from wear-and-tear, increase risk of injury and disability which compromise productivity, and reduce energetic investments in somatic maintenance given constrained energy expenditure. Constraints intensify when nutrient supply is limited and surplus energy is directed toward pathogen defense and reproduction, as occurred throughout hominin evolution. This paper reviews evidence linking exposomes to musculoskeletal health in subsistence populations, focusing on effects of physical activity, pathogens, diet, and reproduction. Chronic musculoskeletal conditions are common for humans and possibly prehistoric hominins but rarer in quadrupedal apes. We propose that transition to bipedalism ~6 to 8 million years ago constituted an early “mismatch scenario," increasing hominin susceptibility to musculoskeletal conditions vis-à-vis quadrupedal apes due to changes in mechanical loading environments. Mismatched musculoskeletal traits were not targets of selection because of trade-offs favoring bipedal extractive foraging and higher fertility.

## Teaser

Unique locomotor and life history demands place humans at elevated risk of chronic musculoskeletal conditions.

## INTRODUCTION

Human aging in contemporary small-scale subsistence populations is characterized by a lack of chronic noncommunicable diseases that are leading causes of mortality worldwide, including cardiovascular disease, Alzheimer’s disease, and type 2 diabetes (*1*–*3*). It has been recognized for decades that reduced life expectancy per se is insufficient to explain why older adults in subsistence populations reliably avoid these common killers (*4*–*6*). Habitually high physical activity levels undoubtedly contribute to robust cardiometabolic health and aging profiles, but what about musculoskeletal (MSK) health and aging? On one hand, early-life exposure to activity-based mechanical stimuli contributes to development and maintenance of structurally resistant MSK tissues, minimizing their risk of deterioration with age. On the other hand, high activity levels can reduce energy allocations to somatic maintenance (*7*), damage MSK tissues via mechanical wear-and-tear (*8*), and create opportunities for traumatic injury and disability (*9*), further increasing demands for tissue repair. Physiological responses to physical activity interact with evolved biological and local socioecological factors that may constrain pathways to healthy MSK development and aging. The human MSK system is unique among primates and mammals, meriting special attention when addressing key evolutionary questions which are explored here: To what extent are human MSK traits a coevolved package of adaptations versus a combination of adaptations and byproducts? How do environmental and lifestyle factors influence MSK system responses to high physical activity levels? What are common lifestyle risk factors for MSK health conditions beyond physical inactivity, consumption of highly processed energy-dense diets, and other risk factors such as excessive drug use that were relatively uncommon in human evolutionary history? And are chronic MSK diseases inextricably linked to chronic noncommunicable diseases of other organ systems, as in higher-income populations where upstream disease risk factors including obesity and metabolic syndrome are shared and themselves intercorrelated?

## MSK CONDITIONS ARE ANCIENT OUTCOMES OF EVOLVED HUMAN BIOLOGY AND THE EXPOSOME

Chronic MSK health conditions are commonly experienced by modern humans and are the most debilitating and costliest conditions worldwide (*10*). Low back pain (LBP) is the top-ranked contributor to global disability (*11*). LBP causes ~50% more disability today than just 25 years ago, and while this increase over time is observed across all ages, it is pronounced for working-age adults. Knee osteoarthritis (OA) is another common chronic MSK condition and has increased in prevalence in contemporary populations by ~100% relative to historic and prehistoric populations (*12*). Incidence of other burdensome MSK conditions including osteoporotic hip and other fractures increased throughout the second half of the 20th century (*13*), but in the past few decades, age-adjusted rates have either increased, plateaued, or decreased in some regions. Increasing osteoporotic fracture rates over time may be due to accelerated rates of bone loss in contemporary relative to ancestral populations (*14*). Together, these findings implicate lifestyle and environmental factors as key inputs influencing human MSK health and aging. In what follows the latent term “exposome” is used as shorthand to refer to the suite of external and internal exposures that individuals experience throughout life. The concept of the exposome, now routinely used in epidemiology, gerontology, and public health, is holistic in the sense that it subsumes lifestyle, environmental, and physiological factors and their interactions over the life course, providing a framework for understanding functioning of interconnected muscular, skeletal, and other organ systems.

Chronic LBP, OA, osteoporotic fracture, and other MSK conditions including bunions, flat feet, hammer toes, and plantar fasciitis are all hypothesized to be “mismatch diseases” (*4*, *15*). Many inherited traits were favored by natural selection to promote biological fitness in distinct ancestral environments, and mismatch diseases can manifest because evolved biology is inadequately adapted to novel environments (*6*). The mismatch hypothesis of disease, now a central guiding hypothesis in evolutionary medicine, posits that the extent and relatively rapid rate of exposome change in our lineage made some conditions more common and severe over time, accelerated age of onset of those conditions, and yielded novel risk factors that were previously rare or absent. Our ability to evaluate these predictions is strongly constrained by the fragmentary and unrepresentative nature of prehistoric samples and the fact that many risk factors in the past cannot be directly observed. While focusing on contemporary small-scale subsistence populations does not generate a perfectly representative view of the past, in the aggregate, such populations do share with past populations more exposome features—including higher physical activity levels and pathogen burdens, relatively unprocessed diets, and natural fertility regimes—compared to well-studied higher-income populations with lower fertility and greater energetic surpluses. Collaborations with subsistence populations (e.g., foragers, horticulturalists, agriculturalists, pastoralists, and societies with mixed economies including forager-horticulturalists) offer potential to generate high resolution, multidimensional longitudinal data linking exposomes to health throughout life. Furthermore, many subsistence populations are undergoing socioeconomic, epidemiological, and demographic transitions, creating further opportunities for testing predictions from the mismatch hypothesis, even if no single population perfectly represents or is matched to the past.

Exposome changes create opportunities for mismatches, but precisely when and under what conditions “novel” exposomes emerged in our lineage is unclear. A common mismatch scenario in the literature attributes as a starting point transition from foraging to farming beginning ~10 to 15 thousand years ago (kya) (*6*). This transition was geographically and temporally variable and accompanied by major changes in mobility, disease ecology, diet, fertility, and social organization, although human genetic composition has changed relatively little since the early Holocene [but see, e.g., (*16*)]. Comparisons between hunter-gatherers and agriculturalists often reveal among the latter, particularly early agriculturalists, decreased body size and skeletal robusticity (i.e., strength relative to body size) and increased skeletal markers of nutritional stress and infectious disease (*17*–*22*). Another mismatch scenario attributes as a starting point the more recent transition toward more efficient production beginning in the 18th century during the Industrial Revolution. Other scenarios emphasize even more recent transformations (i.e., “post-industrialization”) either in food processing techniques, the public health sec tor including water supply improvements or discoveries of antibiotics (*3*), or in digital technology over the past few decades. Recent exposome changes have obviously yielded many survival benefits but may also underlie the increasing burden of many chronic noncommunicable diseases. Studies of contemporary subsistence populations highlight the potential for exposome changes, even within a single generation, to interact with evolved metabolic phenotypes and increase susceptibility to knee OA due to greater accumulation of abdominal adiposity from chronic positive energy balance (*23*).

We propose that transition to bipedalism beginning ~6 to 8 million years ago (Mya) constituted an early mismatch scenario, increasing hominin susceptibility to certain chronic MSK conditions vis-à-vis quadrupedal apes due to changes in mechanical loading environments [cf. (*24*)]. While the hominin fossil record is fragmentary and subject to biased preservation, several chronic MSK conditions, especially those targeting the vertebral column, appear to be more common and severe in early hominins compared to quadrupedal apes. Facet joint OA is observed at least 3.6 Mya in *Australopithecus afarensis* (*25*), as is marked thoracic kyphosis and vertebral disc degeneration ~3.2 Mya (*26*). These conditions cause LBP and difficulty walking and carrying or lifting objects among modern clinical patients (*27*), who may provide indirect insights into lifeways of past hominins. Spinal OA and juvenile kyphosis appear to be prevalent among fossil hominins (*28*). Spinal pathologies more generally are relatively uncommon in quadrupeds including chimpanzees and gorillas (*24*, *29*, *30*). Other great apes can experience some degenerative MSK conditions, but their prevalence and severity are markedly lower than that of humans (*30*). Spontaneous vertebral fractures have not been observed in other apes, even in individuals with severe osteopenia (*31*). Vertebral deformities including fractures are, however, regularly observed in diverse hominin skeletal samples (*26*, *32*–*39*). Fatigue fracture-induced spondylolysis appears to be another condition that uniquely affects humans as primates (*40*).

Early hominins inherited morphological traits that may have been more suitable for meeting functional demands of a largely quadrupedal and semi-arboreal lifestyle characterizing their primate ancestors (*41*). We propose that some inherited traits that were advantageous or neutral in ancestral environments became mismatched following transition to an increasingly bipedal and terrestrial mechanical loading environment. Mismatched traits may not have been targets of strong selection after changes to the mechanical loading environment because of trade-offs ultimately favoring bipedalism. Although hominin MSK biology changed dramatically with transition to bipedalism to accommodate unique mechanical demands of orthograde posture, locomotion, and extractive foraging with regular tool use (*42*–*44*), mismatched traits may still be present in contemporary humans. It has been hypothesized that humans face unique risks of cartilage deterioration and OA for “under-used” joints due to a mismatch between evolved primate joint movement capacities and needs from bipedal mechanical loading regimes (*41*). Whereas other great apes fully use joint movement ranges during tree climbing, brachiation, and quadrupedal locomotion, humans do not use complete movement ranges for glenohumeral and other joints. Others have similarly hypothesized that OA results from a mismatch between evolved joint structure and functional demands of bipedalism but have instead proposed as a primary mechanism mechanical overload from excessive and repetitive stresses (*45*). Bipedal walking places loads on our hindlimbs that are otherwise distributed across hind and forelimbs among quadrupeds. According to this logic, derived morphological changes eventually accompanying transition to bipedalism rendered some joints (e.g., facet and knee joints) poorly equipped or “under-designed” to meet functional demands, increasing risk of joint structural degeneration. Precisely when and under what conditions specific ancestral or derived traits were mismatched remains unclear, and it is possible that multiple mismatches occurred at different times and for different MSK traits. More recent exposome changes (e.g., promoting chronic positive energy balance and obesity) can either exacerbate effects of MSK traits that were already mismatched earlier in hominin evolutionary history or produce distinct mismatches that were not previously associated with transition to bipedalism.

Given hominin variability in morphology, locomotion, life history traits (e.g., adult life span), and exposomes from at least ~4 Mya until 40 kya (*46*), the extent to which hominin survival and reproduction were hindered by the aforementioned and other specific MSK conditions is not apparent or readily discernable from any single species. But an early mismatch scenario—millions of years before the emergence and spread of plant and animal domestication—entails that some chronic MSK conditions became relatively common and severe during hominin evolution vis-à-vis quadrupedal apes. We propose that the rise of these MSK conditions long preceded that of many other chronic noncommunicable conditions (e.g., coronary artery disease) which are now common globally, reliably cooccur with several chronic MSK conditions, and share upstream disease risk factors including physical inactivity, obesity, and metabolic syndrome that are themselves intercorrelated (*4*).

In what follows, evidence from contemporary small-scale subsistence populations linking the exposome to MSK health over the life span is reviewed, with a focus on effects of four exposome features that were more salient over the vast majority of human evolutionary history: (i) high physical activity–based mechanical loading from subsistence effort; (ii) high pathogen burden; (iii) variable nutrient intake including potential for limited micronutrient supply; and (iv) relatively early and rapid reproduction characteristic of natural fertility regimes. There is more focus on bone than other MSK tissues because bone is generally better studied. Moreover, bone is a remarkably dynamic tissue that adjusts its structural properties to meet mechanical and metabolic demands across heterogeneous conditions. Its adaptive plasticity is central to evolutionary life history trade-offs. There is also a disporoportionate focus on Bolivian Tsimane forager-farmers with whom we have worked since 2004 as part of the Tsimane Health and Life History Project (*47*). Tsimane are a valuable case because we have detailed population-representative panel data on their exposomes, health, and aging. Tsimane are not a “premodern” population. They are neither “pure” hunter-gatherers nor agriculturalists, and they differ in important ways from ancestral populations in terms of mobility, disease exposures, diet, and fertility. They live in a humid tropical forest, most of the calories in their diet come from a handful of domesticated plants, and their total fertility rate is higher than most natural fertility populations (*48*). We should thus not expect Tsimane, or any single contemporary population, to represent the diversity characterizing past and present small-scale subsistence populations. In the next section, the unique mechanical stresses placed on the human MSK system in subsistence economies characterized by strenuous physical labor are discussed. Then, in light of evolutionary life history trade-offs, constraints on MSK tissue growth and maintenance under high activity regimes, recurring exposure to diverse pathogens, and variable nutrient intakes are discussed. Afterward, the extent to which mobilization of skeletal calcium reserves in response to obligate demands of gestation and lactation promotes maternal mineral depletion across varying fertility and nutrient regimes is considered. Future research directions for studying variation in MSK functioning in our lineage are discussed in the conclusion.

## MECHANICAL LOADING FROM SUBSISTENCE EFFORT, TISSUE WEAR-AND-TEAR, AND MSK PAIN

Compared to most mammals, humans have distinctive physical activity patterns and derived MSK and other traits promoting endurance (*49*, *50*). Bipedal locomotion reduces energetic costs of walking and likely increased early hominins’ ability to travel between widely dispersed food sources (*51*, *52*). Contemporary foragers and forager-farmers walk about three to four times more per day than chimpanzees and bonobos and >15 times more than other apes (*53*). Despite walking more efficiently for a given size and distance, because humans walk so much in the quest for energy-rich nutrients, we incur higher daily energy costs from subsistence than other apes—both absolutely and as a proportion of total energy expenditure (TEE) (*54*). Humans were likely not exposed to long periods of physical inactivity until recently in evolutionary history, particularly since the Industrial Revolution. Hunter-gatherers, even while resting, engage in low-intensity muscle activity because rest regularly occurs in postures such as squatting or kneeling instead of sitting in chairs. The latter reduces mechanical and energetic demands of supporting the body (*55*).

Inputs from mechanical stimuli including internal tissues, gravitational forces, and ground reaction or other forces from physical activity are required for growth and maintenance of MSK tissues (*56*–*59*). Mechanical loading causes structural deformation of tissues (i.e., “strain”), which respond to several features of the loading environment: number of loading cycles, the strain rate, magnitude, frequency and distribution, and the rest-recovery period. These features interact with each other and with other factors (e.g., genomic and anthropometric) to influence tissue structure as indicated by many empirical studies of nonhuman animals, theoretical models, human exercise interventions, and studies of competitive athletes and clinical and other human samples—usually from higher-income nations. As would be expected of a flexible organ system composed of dynamic parts which can adjust to changing conditions, MSK tissue responses to mechanical loading are variable, depending on the types of weight-bearing activity and muscle function. Few quantitative estimates of naturalistic mechanical loading environments are available in subsistence societies. We lack data, for example, on strain frequency and intensity while carrying objects [cf. (*60*)] and offspring varying in mass and while performing diverse subsistence tasks. Among Mexican Tarahumara subsistence farmers, comparisons of minimally versus conventionally shod individuals show among the former stiffer longitudinal arches (*61*). Minimally shod Tarahumara also have larger abductor hallucis and abductor digiti minimi muscles and stiffer longitudinal arches than conventionally shod Americans (*62*). Footwear is a fairly recent human innovation dating to at least 6.5 to 9 kya that provides mechanical protection from the substrate and insulation (*63*). In altering the distribution and reducing the magnitude of ground reaction forces, modern shoes may weaken foot muscles, reduce foot stiffness, and increase risk of flat feet. Purported MSK mismatch diseases of the foot (e.g., flat feet, bunions, and hammer toes) may largely be attributable to specific and fairly recent changes in footwear, whereas risk factors for other purported MSK mismatch diseases (e.g., OA) appear more multidimensional including mechanically induced tissue damage, local inflammation secondary to mechanical damage, and chronic low-grade systemic inflammation.

Humans often perform repetitive low force tasks such as prolonged walking on irregular surfaces (*64*, *65*) or less repetitive high force tasks such as lifting heavy carcasses or wooden building materials, without experiencing significant tissue injury. Certain types of mechanical loading strengthen MSK tissues (*12*, *58*, *66*), whose responses to applied forces are interdependent and facilitated by endocrine-paracrine cross-talk. Routine early-life exposure to vigorous and dynamic loading can contribute to development and maintenance of structurally resistant tissues, minimizing their risk of deterioration with age.

**Fig. 1.**
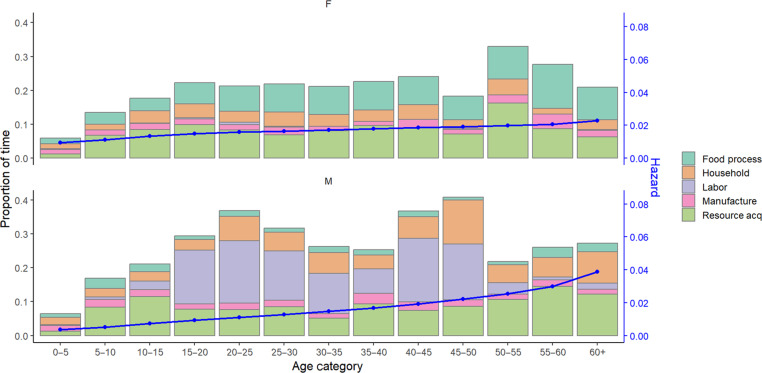
Opportunities for and risks of accidental self-laceration among Tsimane by age and sex. Opportunities for laceration are estimated using time allocation data. Stacked bars show proportion of time at risk of self-laceration (*n* = 55,035 observations of 904 individuals across six villages) from either food processing (e.g., butchering), other household tasks (e.g., sharpening knives or clearing trails with machetes), labor (e.g., building a house or boat, tree chopping, or clearing brush in fields), tool manufacture including children’s play with machetes, and resource acquisition (e.g., harvesting with knives or machetes and hook-and-line fishing). Activities for which there was uncertainty over whether one faced self-laceration risk were omitted from the risk set. Risks of laceration are estimated using a hazard function based on retrospective self-reports among a different Tsimane sample collected contemporaneously as the time allocation data (*n* = 388 individuals aged 11 to 75 years across 16 villages). The hazard function is modeled separately for each sex using the bshazard package in R, stratifying by episode number to account for repeated lacerations within individuals over time. The blue line shows the mean hazard for each 5-year age interval. F, female; M, male.

Yet, strains below mechanical failure thresholds also routinely cause tissue damage (*67*), manifesting, for example, as microcracks in bone or calcified regions of cartilage (*68*). Tissues are constantly damaged and repaired, but damage can accumulate if there are insufficient resources (e.g., energy and time) for repair. This may be common in subsistence populations due to the combination of habitually high physical activity levels relative to consumption, work that can involve repetitive high strain magnitudes, and energetic demands of immune activation from frequent infection and high fertility. In addition, work-related traumatic injuries (e.g., from fractures, contusions, and lacerations) can cause prolonged disability and further increase demands for tissue repair while compromising productivity (*9*, *69*). Compared to more sedentary sex-matched controls from Los Angeles and using directly comparable computed tomographic (CT) methods, age-standardized prevalence of thoracic vertebral fracture is 3.3 times higher for Tsimane men (36% versus 11%) and two times higher for Tsimane women (18% versus 9%) (*70*). In pooled analyses of Tsimane men and women aged 40+ years, neither age nor thoracic vertebral body bone mineral density [BMD; i.e., the mean of three consecutive vertebrae (T7 to T10 range) excluding their vertebral shells] strongly predicts thoracic vertebral fracture, suggesting an important role of blunt trauma in fracture etiology. Work-related trauma also commonly causes injuries to soft tissues including muscles, ligaments, tendons, and cartilage: 80% of Tsimane report ever experiencing an accidental self-inflicted laceration severe enough to cause disability (i.e., preclude any work; maximum days disabled = 300; median = 7). Tsimane laceration risk gradually increases with age, partly because of age-related increases in time spent at risk during subsistence work (Fig. 1).

Tendons, ligaments, and cartilage have more limited self-repair capacity than skeletal muscle and bone. Bone remodeling is critical to remove damage because bone is roughly twice as heavy as other tissues, and there is selection to minimize bone mass while maintaining its structural integrity (*71*). Remodeling allows bone to flexibly alter its architectural properties for meeting mechanical demands while supporting its immune, endocrine, and reproductive functions (see below). Even a few dozen loading cycles can require hours of recovery for bone cells to restore peak responsiveness (*72*). Repetitive work-related mechanical stress can accelerate senescence of load-bearing joints, as suggested by the relatively high rates of often painful OA in the distal interphalangeal joints of textile mill workers, in the knees of those with physically demanding occupations entailing frequent knee bending, in the hips of farmers regularly lifting heavy weights for prolonged periods, and in joints that are rarely affected by OA such as elbows, wrists, and metacarpal phalangeal joints among jackhammer operators [see citations in (*8*)]. Excessive and repetitive overloading is also posited to be a primary cause of human tendinopathy (*73*).

**Fig. 2.**
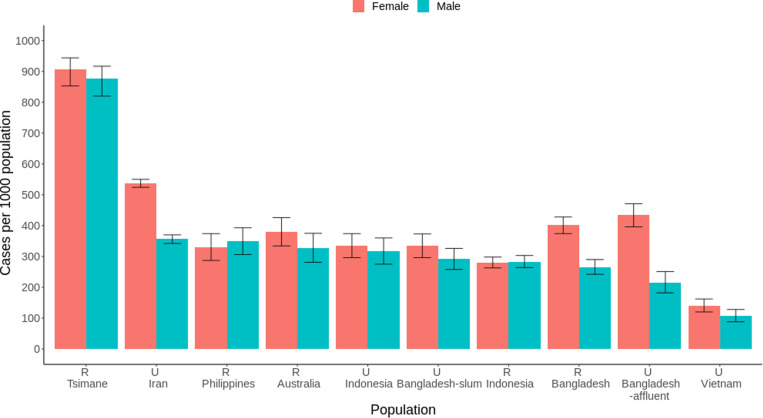
Current musculoskeletal pain prevalence (age-standardized using the direct method) by sex and population. The 95% confidence intervals are exact binomial. Populations are shown in descending order of male prevalence. Estimates are derived using an identical minimum age of 15 years across populations. Reproduced from (*8*). R, rural sample; U, urban.

As would be expected if repetitive work-related mechanical stress involving rife potential for injury increases demand for tissue repair, Tsimane exhibit high MSK pain prevalence in cross-cultural perspective (Fig. 2). Neurophysiological pain mechanisms are highly conserved in mammals (*74*), and certain pain behaviors are shared across species. The dorsal horn of the spinal cord contains the first synapse in pain pathways, and descending control of spinal nociception originates from many brain regions and circuits affecting pain experience. The characteristic aversive pain experience produces a coordinated set of cognitive and behavioral responses including focused attention, vigilance, and flight, which jointly prioritize self-preservation (*75*). Pain thus serves functions of somatic maintenance and defense: It minimizes tissue damage by limiting movement that could cause further damage, facilitates tissue repair by motivating escape from harmful situations, and motivates learning to avoid future damage. However, in subsistence economies, the ability to avoid future damage or escape harmful situations can be limited (e.g., see Fig. 1).

The vast majority of self-reported Tsimane MSK pain attributions [i.e., >85% of 729 attributions across different anatomical locations within individuals (*n* = 285 individuals)] are from subsistence work and work-related accidents including falls from either trees, dugout canoes, or slippery footbridges while carrying heavy loads (*8*). Bites, stings, and attacks from snakes, stingrays, potential prey, and a large variety of insects are also burdensome, and infections from those assaults are not uncommon. The most common work-related pain attributions are from, in descending order of frequency, horticulture (29% of all work-related pain attributions), excessive load carrying (25%, which includes carrying cultigens or carcasses), other food production (i.e., hunting, foraging, and fishing; summed = 20%), and long-distance walking (17%). These four work subcategories, which are not always mutually exclusive, are also the most common attributions for chronic pain. Age-standardized prevalence of Tsimane chronic pain (defined as lasting either ≥3 or ≥6 months) is within the range of variation observed elsewhere (*8*), but these comparisons are limited to higher-income populations since few chronic pain studies exist in small-scale subsistence populations. Compared to other work-related pain attributions, those from hunting or foraging show higher maximum and median pain durations (max = 1825 days, median = 30). Tsimane pain is most often reported in the lower back, but shoulders and knees are also frequently affected, heavily loaded during subsistence tasks, and common sites of tendinopathies among humans more generally. Most Tsimane pains are experienced while they are working, even for younger individuals, suggesting that damaged MSK tissues fail to completely heal before resumption of physically demanding activities, which can cause additional damage and pain vulnerability. While sleep can facilitate recovery, Tsimane do not sleep more than individuals in post-industrialized populations (*76*), and many injuries require at least several weeks to heal (*8*). Tsimane pain is also more common with age, as in many other subsistence populations, likely due to declining physical condition and greater cumulative imbalances between tissue damage and repair from recurrent mechanical stress and injury. Sex differences in Tsimane pain prevalence are relatively weak (Fig. 2), which is not unexpected since both sexes routinely walk long distances, participate in strenuous horticultural and other work, and experience work-related hazards that are not limited to falls, including sunburn and puncture wounds from thorns while walking barefoot.

What broader inferences may one draw about MSK pain experience during human evolution from the detailed Tsimane case? One cannot obviously make direct inferences about pain experience of prehistoric populations, and earlier hominins vary greatly in their morphology, locomotion, life span, and mode of subsistence. Nevertheless, the finding that subsistence work including long-distance walking, foraging, hunting, and farming underlies much acute and chronic pain may generalize across diverse human populations and time periods given our community-based sampling, participants’ limited pain prevention and treatment options, and the mixed Tsimane subsistence economy. We surmise that chronic pain of the lower back and possibly other anatomical regions was not uncommon before dietary reliance on intensive agriculture and that transition to orthograde posture and associated changes in compressive loading patterns made bipedal hominins more susceptible to MSK pain from recurrent mechanical stress. Some of the greatest compressive spinal loads occur when weights are carried in front of the body (*77*), and for many activities that are commonly performed across subsistence modes (e.g., neutral standing, standing or walking with weight, mild trunk flexion and extension, and lifting objects above the head), the greatest spinal compressive loads are generated in the thoracolumbar region. Recurrent mechanical stress and traumatic accidents that are inextricably linked to subsistence work constrain the extent to which even highly conserved adaptive pain mechanisms minimize tissue damage and promote repair. Regular exposure to infectious diseases and limited nutrient supply (discussed in the next section) intensify these constraints since somatic defense and maintenance require time and energetic resources. Degenerative changes in MSK system components such as joints that often cause pain result largely from mechanical demands throughout life, and while some bioarchaeological studies report a higher prevalence of OA and osteophytosis among agriculturalists than hunter-gatherers, other studies report either no difference in prevalences by subsistence mode or higher prevalences among hunter-gatherers than agriculturalists [reviewed in (*78*)]. There is also bioarchaeological evidence that traumatic injury risk (proxied by postcranial fracture prevalence) is lower among low-intensity agriculturalists relative to either hunter-gatherers or high-intensity agriculturalists (*79*). Moreover, it is possible that variation across subsistence modes in interpersonal violence [cf. (*80*)] contributed to differences in MSK pain prevalence across populations. But there is substantial variability in rates of physical aggression within subsistence regimes, and among Tsimane, only eight percent of all pain attributions are from interpersonal conflict or competition. Most Tsimane interpersonal conflicts precipitating physical violence and pain occur within families (*81*) rather than between families or other groups.

Generally speaking, in response to recurrent mechanical stress and potential for tissue damage, natural selection is expected to have shaped mechanisms to respond adaptively by altering pain thresholds and salience. Adaptive responses to recurring painful stimuli can, in theory, be characterized by increased pain sensitivity (consistent with the “smoke detector principle”) or decreased pain sensitivity (e.g., during callus formation from recurring skin abrasions) (*82*). A key evolutionary question is to what extent changing exposomes generate adaptive alterations or deleterious side effects, which can be vulnerable to runaway positive feedback, whereby lower pain thresholds increase pain risk.

## CONSTRAINED MSK FUNCTION ACCOMPANYING ACTIVE LIFESTYLES: ROLES OF PATHOGENS AND DIET

Evolutionary life history theory posits that energetic limitations intensify trade-offs between investments in competing demands of maintenance, growth, and reproduction. All else equal, high physical activity levels reduce energy availability (i.e., calories consumed − calories expended) for essential physiological functions, imposing constraints on somatic repair capacities (*83*). For humans and many other organisms, physical activity is the most variable component of TEE, and it can also be the largest component under high activity regimes. Experimental and observational TEE studies suggest that organisms adapt to increased activity levels via behavioral and physiological energy-sparing mechanisms that maintain TEE within a confined range (*7*). TEE increases with physical activity at lower activity levels, but then TEE plateaus at higher activity levels. Reductions in energy expenditures on functions unrelated to activity including tissue repair may underlie symptoms associated with overtraining in athletes (e.g., fatigue) and help explain the high Tsimane MSK pain prevalence cross-culturally (Fig. 2).

High pathogen burden constrains energy availability and is a common exposome feature of subsistence populations. Immune responses can be energetically costly due to demands of fever and increased protein synthesis during the acute phase response (*84*). Great apes including humans show stronger and less specific leukocyte responses than African and Asian monkeys during the first 24 hours after bacterial and viral stimulation (*85*), despite potential costs of greater energy expenditure and collateral damage. In human subsistence populations, individuals experiencing high pathogen burden prioritize immune function over somatic growth when less energy is available (*86*–*92*). The immune system, like the MSK system, relies on environmental inputs to guide its development and function, and pathogen exposure is a major force shaping human genetic variation (*93*, *94*). Human immune regulatory pathways evolved in a context of repeated and diverse pathogenic exposures (*95*). Whether human energy regulation patterns evolved to support either chronically activated immune systems because of repeated infectious exposures or immune systems that are frequently activated and deactivated is debated (*96*). Among Ecuadorian Shuar forager-horticulturalists, children’s resting metabolic rate (RMR) is higher than children’s RMR in post-industrialized populations, and Shuar excess RMR is associated with higher blood concentration of total immunoglobulin G antibody (*97*). Similarly, Tsimane RMR is higher than predicted by standard equations relying only on age and anthropometrics that were developed in other populations, and after adjusting for potential confounders, Tsimane excess RMR is associated with elevated leukocytes and the presence of intestinal helminths (*98*). Intestinal parasites are common in subsistence populations (*99*, *100*), have coexisted with humans for millennia (*101*), and modulate human immune responses in ways that optimize their own survival and reproduction (*102*). Studies of children in a rural Gambian farming population document correlations between gastrointestinal pathogen exposure, small intestinal mucosal damage, impaired nutrient digestion, absorption and barrier functions of the intestine, and growth faltering (*87*, *88*, *103*). Pathogen exposure can also contribute to growth faltering across generations: Maternal infections during pregnancy are associated with reduced infant birth weight [see citations in (*104*)]. Gambian infants are smaller than age-matched British infants in terms of weight, height, upper arm circumference, and radial bone width (*105*). Gambian infants have 11% lower radial bone mineral content than British infants in unadjusted analyses, and this difference declines to 6% after adjusting for weight, height, and bone width. Maternal infection during pregnancy is one potential mechanism underlying these population-level differences in bone structure. Early-life skeletal differences between these populations persist into older adulthood (*106*) but may not necessarily promote higher Gambian fracture risk if, despite its reduced mineral content, bone tissue retains a sufficient safety factor to withstand applied loads.

Recruitment of immune cells underlying pathogen defenses involves production of reactive oxygen species (ROS), which modulates bone cell function and generates oxidative stress when ROS production is high (*107*). ROS production is involved in mineral tissue homeostasis and contributes to bone remodeling by promoting resorption via osteoclast activity. Bone resorption promotes decarboxylation of osteocalcin (the second most abundant protein after collagen in bone extracellular matrix), decreasing its binding affinity for hydroxyapatite and facilitating its release into circulation and subsequent hormonal action. Decarboxylated osteocalcin has various functions including regulating glucose homeostasis in the pancreas, liver, and muscle, reducing visceral and liver fat accumulation, and regulating the acute stress response (*108*). ROS production also initiates bone loss by inducing apoptosis and reducing osteoblast activity. Certain proinflammatory cytokines down-regulate production of bone matrix proteins within osteoblasts including osteocalcin (*109*). Lymphocytes also promote ROS production, and lymphocytes are elevated among Tsimane relative to Americans and Europeans because of the high Tsimane pathogen load. Tsimane lymphocyte counts are moderately stable within individuals over time (intraclass correlation coefficient = 0.5; mean number of years between measures = 2.1) (*96*). Oxidative stress may be involved in MSK system pathogenesis including osteoporosis, bone tumor development, and joint inflammatory diseases including rheumatoid arthritis. Thus, even under conditions of high mechanical loading from physically active lifestyles, potential compensatory mechanisms underlying bone functional adaptation (e.g., periosteal expansion with aging) can be mitigated by trade-offs favoring immune function.

Bone marrow is a key immune system component due to its role in producing immune cells. Investing in the growth of bone constituents like marrow can therefore simultaneously be compatible with investing in somatic maintenance—contrary to a standard tenet of evolutionary life history theory stipulating that investments in growth and maintenance necessarily trade-off against each other. Minerals such as calcium that are stored in bone also provide key energetic substrates for supporting immune function (*110*). Once calcium is liberated from the skeleton via immunological or endocrine mediators (e.g., interleukin-6 or parathyroid hormone), it is used by innate and acquired immune systems in cell activation, effector functions, and gene expression. In higher pathogen contexts, one might expect (all else equal) diminished osteogenic responses to high physical activity levels due to mobilization of skeletal mineral stores and proteins from bone’s crystal structure for supporting increased energetic demands of immune activation (*111*). Consistent with this expectation, after adjusting for potential confounders, Tsimane adult leukocyte count is inversely associated with ultrasound-derived proxies of calcaneal stiffness [i.e., speed of sound (SOS; in meters per second, reflecting ultrasound wave velocity for a given heel width) and broadband ultrasound attenuation (BUA; in decibels per megahertz, reflecting wave attenuation); calcaneal BMD can be estimated from a linear combination of SOS and BUA; see (*111*, *112*) for additional details]. Leukocyte count is moderately positively correlated within Tsimane adults over time [partial *r* = 0.5, controlling for years between measures (mean number of years between measures = 2.4)], and adults with persistently low counts have higher adjusted calcaneal stiffness proxies (6 to 8%) than adults with at least one high count. By volume, the human calcaneus is >90% trabecular bone, which is more porous, metabolically active, and prone to age-related loss than cortical bone. Calcaneal trabecular structure of the genus *Homo* is more anistropic and less dense than that of *Pan*, *Gorilla*, and *Pongo*, although immune activation per se may not necessarily inhibit bone tissue functionality amidst changes to its structure. While it is widely recognized that morbidity inhibits growth (*113*, *114*) and that immunological mediators regulate osteoblast and osteoclast activity (*115*, *116*), effects of pathogen burden on bone properties and strength have not been well-characterized in humans. Bone is normally a sterile area, but some of the most prevalent skeletal diseases are due to pathogenic actions on bone including inflammatory processes stimulating bone degradation, inhibition of bone matrix synthesis (*117*), and destruction of noncellular bone components by liberation of acids and proteases—as in the case of dental caries, which are common among Tsimane (*118*) and Hadza foragers of Tanzania (*119*).

Constraints on osteogenic responses to high physical activity levels are exacerbated when supply of micronutrients including calcium is limited. All calcium needed for growing and maintaining bones must originate from the diet, as there are no major calcium reservoirs outside of the skeleton. An adult human body contains 1200 to 1500 g of calcium, of which >98% is stored in the skeleton. Calcium metabolism responds adaptively to nutritional supply and physiological demands including somatic growth, immune activation, gestation, and lactation (see below). Monkeys and nonhuman apes consume much low energy but calcium-rich plant food, and some posit that past hunter-gatherer diets also contained high calcium levels (>1500 mg/day) (*120*). Others claim that hunter-gatherer calcium intakes were lower (<400 mg/day) (*121*). Before animal domestication, no humans consumed dairy products after weaning, and calcium was primarily ingested from uncultivated plants (e.g., roots, leaves, nuts, and fruits) and, to a lesser extent, animal products.

Modern human calcium intake varies widely from 175 to 1233 mg per adult per day (*n* = 74 countries across six continents) (*122*). Tsimane intake falls on the lower end of this distribution at 239 mg per adult per day, with no observed sex differences (*123*). Few Tsimane families (<5%) raise cattle, and cattle owners do not make dairy products. Fish and seasonal fruits (e.g., oranges, grapefruits, and mangos) are principal calcium sources. Tsimane diet mainly consists of cultigens grown in small swiddens, which together comprise >60% of calories. Cultigens are generally a poorer source of calcium than uncultivated plants and contain more acids like phytates and oxalates that bind calcium and reduce its intestinal absorption. Tsimane dietary staples in descending order of their daily total energetic contributions are as follows: plantains [~2 mg calcium per 100 g; (*124*)], rice (4 mg), sweet manioc (16 mg), and corn (7 mg). Relative to Western standards, Tsimane intake of other bone-forming minerals and select macronutrients influencing calcium bioaccessibility are either ample (magnesium: ~525 mg per adult per day; zinc: ~14 mg per adult per day) or high (phosphorus: ~1550 mg per adult per day; protein: ~130 g per adult per day). Tsimane diet contains only trace amounts of vitamin D, but most vitamin D necessary for supporting homeostasis and reproduction is produced in the skin from sunlight exposure (*125*), which is abundant for tropical subsistence populations. Tsimane intake of specific nutrients may differ in important ways from other subsistence populations, but human dietary diversity is pronounced and varied markedly throughout evolutionary history. The low Tsimane calcium supply is nevertheless instructive for understanding how diet interacts with physiological demands of reproduction to differentially affect MSK outcomes by sex, which is discussed next.

## MATERNAL DEPLETION OF SKELETAL MINERAL RESERVES UNDER NATURAL FERTILITY REGIMES

Natural selection prioritizes maximizing earlier reproduction over longevity, so organisms increase fertility at the expense of later life survival (*126*–*128*). Energetic investments in reproduction are expected to trade-off against investments in somatic maintenance (*129*). Modern human life histories are remarkable in comparative cross-species perspective because we simultaneously exhibit extended adult life spans and high fertility rates, and we invest substantially in multiple dependent offspring (*54*, *130*, *131*). But what are the implications of this costly life history strategy for MSK function and health in the short and longer term, particularly for women given their disproportionate investments in gestation and lactation? Since the 1960s, “maternal depletion syndrome” was identified as an outcome of interactions between exposome features including repeated closely spaced pregnancies and bouts of lactation, limited nutrient supply, and high pathogen burden. Jelliffe and Maddocks (*132*) focused on small-scale farming populations with natural fertility and low protein intakes by Western standards. Jelliffe and Maddocks wrote: “Probably the earliest recognized, classical maternal depletion syndrome resulting from the increasing and cumulative nutritional stresses of successive pregnancies and lactation was osteomalacia due to drainage of calcium and vitamin D from the woman’s body…(1964:435).” To date, few studies have quantified the extent of this drainage in a cumulative fashion across varying ecological conditions and fertility regimes.

Prior maternal depletion studies in subsistence populations generally focus on maternal weight and adiposity as outcomes, which in response to short-term changes in energy balance fluctuate more than bone tissue properties. Empirical evidence for maternal depletion is mixed (*70*, *133*), and longitudinal studies of maternal somatic costs in natural fertility populations are scant (*134*). At a proximate level, direct trade-offs between maternal energetic investments in reproduction (i.e., gestation and lactation) and maintenance are expected to manifest in the skeleton because mineral allocations to these competing demands draw from the same skeletal reservoir. We should also expect maternal bone tissue to flexibly adjust to variable metabolic and mechanical demands. Energetic demands of reproduction are substantial in natural fertility populations (*135*), and while fertility patterns are spatiotemporally variable (*48*, *136*), throughout much of our evolutionary history energy surpluses were regularly converted into higher fertility (*137*). Tsimane are characterized by relatively early and high fertility: Mean age at first birth is 19 years (before accrual of peak bone mass), mean interbirth interval (IBI) is 34 months, and the total fertility rate is nine births per postreproductive woman. Greater Tsimane reproductive effort—proxied by earlier age at first birth and shorter mean IBIs—is associated with reduced calcaneal stiffness and thoracic vertebral body BMD as determined by ultrasonography and CT, respectively [ (*70*, *112*); see also (*138*) for a similar analysis using calcaneal ultrasonography among the Shuar of Ecuador]. Tsimane women’s vertebral fracture risk increases with lower vertebral body BMD and shorter mean IBIs even after adjusting for BMD and potential confounders. While Tsimane total fertility rate is higher than most natural fertility populations, parity per se does not reliably predict Tsimane bone properties after adjusting for maternal age and anthropometrics, age at first birth, and mean IBIs. Generally speaking, human peak bone mass is not achieved until the late 20s, and teenage pregnancy and lactation can disrupt maternal growth and bone mineralization for Tsimane and other populations (*139*, *140*). In terms of understanding human health over the life course, trade-offs between energetic investments in growth and reproduction have received less empirical attention than trade-offs between reproduction and maintenance.

**Fig. 3.**
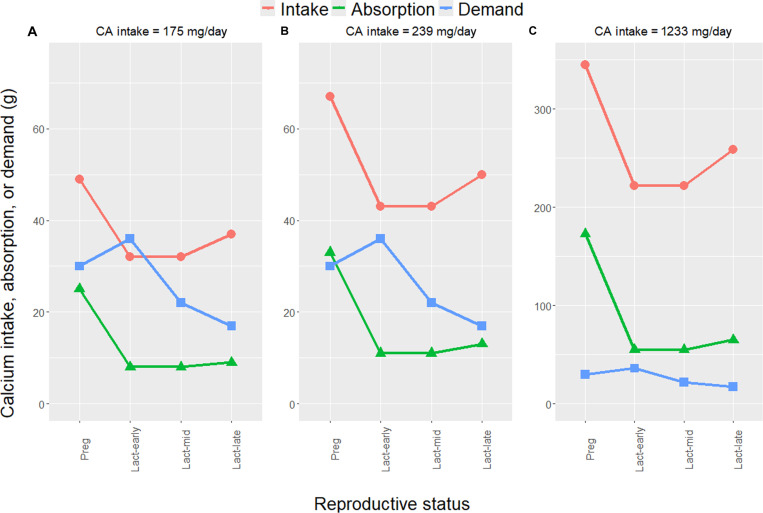
Human female calcium economy (i.e., intake, absorption, and demand; in grams) during a single synthetic reproductive bout composed of gestation and lactation. (**A** and **C**) Minimum and maximum observed calcium intakes, respectively (*122*). (**B**) Tsimane intake (*123*). Calcium absorption is 50% of intake during pregnancy (preg) and 25% during lactation [lact; (*142*)]. Demand consists of fetal skeletal calcium accretion (30 g at parturition for a full-term 3.5-kg neonate) and daily maternal-infant calcium transfers during lactation. Three periods of lactation are shown: early (first 180 days), middle (mid; subsequent 180 days), and late (final 210 days), signifying the decline in daily maternal-infant transfers over time. Estimated calcium transfers during early and middle lactation periods (i.e., 200 and 120 mg/day, respectively) are from (*142*). Late period transfers (81 mg/day) are estimated from two recent meta-regressions: (i) breast milk calcium concentration on days since parturition (*145*) multiplied by (ii) age-specific breast milk intake [see Table 3 in (*189*)]. Inclusion of the late lactation period reflects prolonged breastfeeding typical of natural fertility populations, using as a benchmark the mean Tsimane weaning time of 19 months (*190*). Pregnancy is assumed to last 280 days. Maternal calcium intake is assumed to be constant during pregnancy and lactation [cf. (*191*)]. Calcium absorption efficiency is assumed to be independent of intake and other factors. Maternal calcium demand for maintaining homeostasis is not considered here, and thus, demand is a lower bound estimate.

Maternal physiology responds to greater mineral demands of gestation and lactation by mobilizing skeletal mineral stores (*141*). The human fetal skeleton accretes ~30 g of calcium by term (Fig. 3) of which ~80% is accreted in the third trimester [see (*142*) for a comprehensive review]. Maternal bone turnover increases during gestation, especially during the third trimester, creating a net resorptive state facilitating fetal mineral transfers. While ~25% of dietary calcium intake is typically absorbed in adults, additional calcium demands during gestation beyond those required for maintaining homeostasis are met by a doubling in the efficiency of intestinal calcium absorption from as early as 12 weeks of gestation, mediated in part by 1,25-dihydroxyvitamin D. Greater calcium absorption facilitates its storage in preparation for elevated demands later in gestation and lactation. Analyses of bone turnover markers, BMD, and bone microarchitecture using high-resolution peripheral quantitative CT in higher-income populations suggest that lactating women are in negative calcium balance, especially when breast milk production is elevated (*142*). The peak rate of bone mineral loss during lactation is ~1 to 3% per month, which far exceeds the rate of ~1 to 3% per year in women with postmenopausal osteoporosis. Lactation-induced BMD reductions are pronounced at skeletal sites rich in trabecular bone. High calcium intake does not appear to affect the degree of skeletal demineralization during lactation. For exclusively breastfeeding women, during the first 5 months of lactation, breast milk production rates are similar cross-culturally (*143*). But from 6 months onward, variation in breast milk production increases, as infant breast milk intake declines with complementary feeding. Breast milk composition varies considerably cross-culturally. For instance, among rural Gambian farmers with low calcium intakes (~400 mg per adult per day) and on-demand breastfeeding, mean breast milk calcium concentration throughout lactation is 19% lower than in Cambridge, UK (*144*). Breast milk calcium concentration is independent of maternal age and parity in both populations. In other populations with low calcium intakes, breast milk calcium concentration is similar to that in populations with higher calcium intakes [see references in (*144*, *145*)]. During the first 6 months postpartum (and second 6 months, respectively), ~200 mg/day (and ~120 mg/day) of calcium is secreted into breast milk (*142*). Breast milk calcium concentration declines gradually thereafter based on globally representative random effects meta-analyses (*145*).

Figure 3 shows human female calcium economy (i.e., intake, absorption, and demand; in grams) for a synthetic reproductive bout composed of gestation and lactation. It illustrates the scope for maternal skeletal mineral depletion in the short term during a single reproductive bout by comparing calcium absorption from dietary intake and physiological demand. It also highlights the adaptive plasticity of bone tissue since this maternal skeletal mineral depletion typically does not immediately compromise bone structural integrity. Figure 3 shows three different daily calcium intakes given spatiotemporal variation in human diets: Fig. 3 (A and C) shows, respectively, the minimum and maximum observed human calcium intakes (*122*), and Fig. 3B shows the Tsimane intake (*123*). At the minimum intake of 175 mg per adult per day (Fig. 3A), calcium demand exceeds absorption during gestation and lactation. This negative calcium balance totals 5 g in gestation, which is equivalent to 17% of the calcium content of a fetal skeleton (30 g) and 0.4% that of an adult female skeleton (1200 g). Across the first 6 months of lactation, this negative calcium balance totals 28 g (2.3% of an adult female skeleton). At the Tsimane calcium intake of 239 mg per adult per day (Fig. 3B), absorption slightly exceeds demand by 3 g in gestation, and across the first 6 months of lactation, demand exceeds absorption by 25 g (2.1% of an adult female skeleton). At the maximum intake of 1233 mg per adult per day (Fig. 3C), absorption far exceeds demand across gestation and lactation. Together, these estimates apply to only one reproductive bout, whereas maternal depletion syndrome considers the cumulative somatic effects of multiple bouts (*132*). For this reason, it is necessary to consider maternal calcium economy throughout the entire reproductive life span, which I discuss next.

**Fig. 4.**
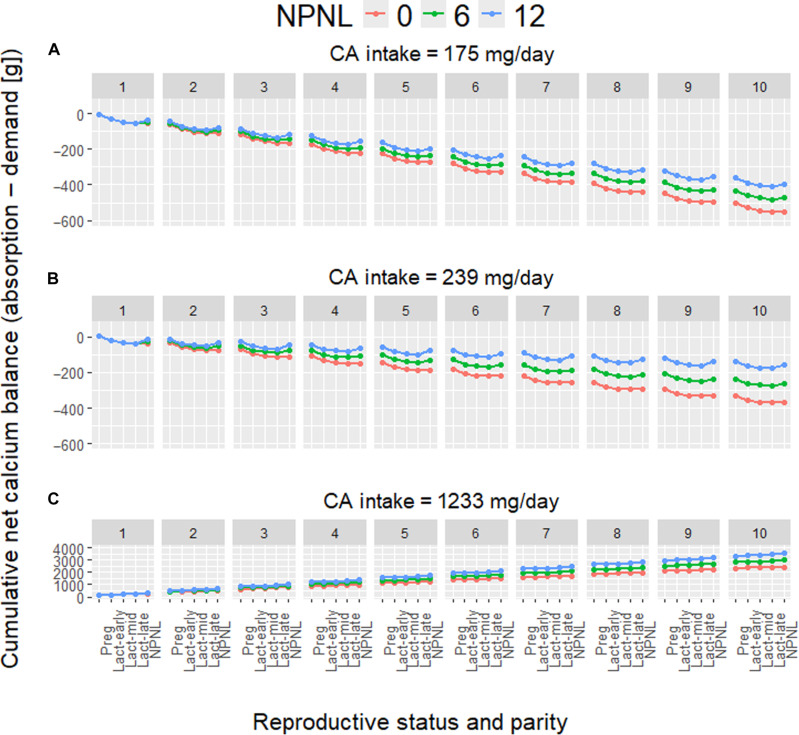
Cumulative net calcium balance (i.e., absorption − demand; in grams) by reproductive status and parity (range: 1 to 10) across variable calcium intakes and periods of maternal recovery following each reproductive bout. Calcium balance is calculated from the running total across reproductive statuses and bouts. (**A** and **C**) Minimum and maximum observed human calcium intakes, respectively. (**B**) Tsimane intake. Reproductive status includes pregnancy, three periods of lactation, and a NPNL phase. The NPNL phase is the duration from weaning until the next conception and is characterized by zero calcium demand from reproduction. Three NPNL phase durations are shown: 0 months (i.e., no maternal calcium recovery from weaning until the next conception) and 6 and 12 months (see text for additional details). Maternal calcium demand for maintaining homeostasis is not considered here, and thus, demand is a lower bound estimate.

Figure 4 shows net calcium balance (i.e., absorption − demand; in grams) in a cumulative fashion across reproductive bouts and statuses within bouts. A running total of the net balance illustrates the scope for maternal skeletal mineral depletion from reproduction in the longer term, independent of the effects of aging. A maximum of 10 live births is shown (miscarriages are omitted), given spatiotemporal variation in human total fertility rates. Figure 4 includes a nonpregnant nonlactating (NPNL) phase following each reproductive bout, spanning the duration from weaning until the next conception. Women can increase their bone mass and mineralization after weaning, potentially reversing at least some of the transient losses incurred during lactation. This restorative capacity partly depends on lactation intensity and duration. For example, among healthy American women, extended lactation (≥6 months) is associated with BMD losses of ~5% at the lumbar spine, with restoration of BMD to prepregnancy values by 12 months postpartum (*146*). In contrast, rural Gambian women, who have closely spaced births and on-demand breastfeeding for approximately 2 years, exhibit incomplete restoration of lumbar spine BMD to prepregnancy values by 12 months postpartum (*147*). Complete BMD restoration for Gambian women may occur only after weaning (*148*). Figure 4 shows three hypothetical NPNL phase durations: 0 months (i.e., no maternal calcium recovery from weaning until the next conception), 6 months, and 12 months. Precise estimates of the NPNL phase duration are lacking in natural fertility populations, and one would expect substantial variability within women across births, between women, and between populations (*136*). The three hypothetical NPNL phase durations shown in Fig. 4 are meant to consider some of this variability without representing any specific population.

At the minimum calcium intake of 175 mg per adult per day (Fig. 4A), after five reproductive bouts and with no NPNL phase following each bout, the net calcium balance reaches a deficit of 276 g (equivalent to 23% of an adult female skeleton). At this same intake, increasing the NPNL phase to 6 months lowers the net deficit to 237 g (20% of an adult female skeleton). Further increasing the NPNL phase to 12 months reduces the deficit to 197 g (16%). These deficits double after 10 reproductive bouts, such that, in the extreme case with no NPNL phase, 552 g of calcium (46%) is depleted from the maternal skeleton. At the Tsimane intake of 239 mg per adult per day (Fig. 4B), after five reproductive bouts and with no NPNL phase following each bout, the deficit reaches 185 g (15%). Increasing the NPNL phase to 6 months reduces the deficit to 133 g (11%). Further increasing the NPNL phase to 12 months reduces the deficit to 78 g (7%). After 10 bouts, in the extreme case with no NPNL phase, 371 g of calcium (31%) are depleted from the skeleton. Thus, at the lower end of the human calcium intake distribution, even small daily per capita increases from 175 to 239 mg can restore maternal skeletal mineral deficits to a similar or greater extent than increases in NPNL phase durations by 6 to 12 months (all else equal). At the maximum intake of 1233 mg per adult per day, there is no maternal calcium deficit whatsoever (Fig. 4C). Even with no NPNL phase following each bout, after five bouts, there is a positive calcium balance of 1219 g—roughly the equivalent of an entire adult female skeleton.

## OUTLOOK AND FUTURE RESEARCH DIRECTIONS

Some hypothesize that the high physical activity levels characteristic of human foragers relative to other great apes promoted selection in our lineage for increased tissue repair and maintenance in response to physical activity (*53*). Compared to nonhuman primates, humans exhibit higher levels of circulating antioxidants (*149*), lower rates of physiological dysregulation (*150*), and higher levels of the steroid hormone dehydroepiandrosterone sulfate, which promotes somatic maintenance (*151*). Greater maintenance of regulatory processes with aging helps explain the long human adult life span relative to other primates, but whether species-level variation in activity levels underlie these physiological and life history differences merits investigation. Tsimane are not representative of all human subsistence populations, but their exceptionally high prevalence of MSK pain (Fig. 2) (*8*) and degenerative arthritis (*70*, *152*) suggests that levels of MSK tissue repair and maintenance are inadequate for meeting functional demands. Constraints on energy expenditure in physically active populations (*83*) coupled with high energetic investments in pathogen defense and limited nutrient supply can inhibit repair and maintenance capacities (*70*, *111*, *112*, *153*, *154*).

These constraints are exacerbated for women given their high energetic investments in gestation and lactation (*129*, *132*, *138*). The timing and amount of reproductive effort have likely affected female MSK health and aging throughout hominin evolutionary history, but reproduction and its interactions with exposome features are often overlooked in paleoanthropological and bioarchaeological studies. A growing literature emphasizes sexual dimorphism in MSK tissue responses to activity-based mechanical loading (*155*), mediated in part by sex steroid and growth hormones, growth factors, and their receptors (*156*). Consistent with this literature, thoracic vertebral body BMD is lower for Tsimane than age-matched American women, whereas for men, no population-level BMD difference is apparent—despite higher Tsimane physical activity levels for both sexes (*70*, *112*). Dietary or other systemic population-level differences (e.g., in pathogen burden) do not easily explain Tsimane women’s lower BMD because such systemic factors should affect both sexes. Female reproductive effort can affect MSK tissues directly [e.g., Figs. 3 and 4; (*70*, *112*)] and indirectly, since gestation and lactation lower mobility and activity-based mechanical loading [cf. (*157*)]. Notably, Tsimane exhibit relatively weak sex differences in MSK pain (Fig. 2), and women’s pain is not associated with proxies of reproductive effort including age at first birth, mean IBI, and parity (*8*). Future research into how exposome features interact differentially by sex across varying environments to influence development, repair, and maintenance of MSK system components will reveal important insights.

One of the greatest exposome shifts in human history occurred with transition from foraging to farming, which was accompanied by extraordinary population growth, expansion, and demographic fluctuations (*158*). Transition to nutritional dependence on relatively few carbohydrate-rich cultigens likely entailed at least in some regions and time periods reduced dietary diversity and intake of protein and micronutrients (*15*, *17*, *121*). Many burdensome infectious diseases afflicting humans proliferated through processes of domestication (*21*, *159*) and with increasing population size and density of early farming communities (*160*). It has recently been proposed that transition from foraging to farming fundamentally changed the life history trade-offs underlying human somatic energy allocations, by increasing investments in pathogen defense and reproduction and decreasing investments in growth and forms of maintenance unrelated to pathogen defense including skeletal remodeling (*104*). According to this model, new niche-specific optima were favored that ultimately led to reduced body size and skeletal robusticity, accelerated onset of reproduction and IBIs, and greater total fertility rate among early farmers compared to their forager predecessors [also see (*161*)].

Consistent with this model, several studies document during transition from foraging to farming greater skeletal markers of nutritional stress and infectious disease [e.g., (*17*, *21*)], smaller adult body size [reviewed in (*104*)], and a general decline in skeletal mechanical competence—in cortical and trabecular bone of the lower limbs in particular (*18*, *162*–*166*). Comparisons of adult skeletal remains of hunter-gatherers and agriculturalists show among agriculturalists accelerated age-related decline in radial bone mineral content (*167*) and reduced femoral strength (*165*, *168*). But if bone tissue retains a sufficient safety factor to withstand applied loads, then these structural changes need not imply increased fracture susceptibility. Reduced investments in skeletal growth and maintenance accompanying farming were traditionally interpreted as reflecting lower levels of activity-based mechanical loading in more sedentary farming communities. But studies of contemporary subsistence populations reveal that activity levels vary substantially within a given subsistence regime, are generally moderate to high among farmers (*169*), and can be even higher for farmers than foragers (*170*, *171*). Children in agricultural societies also generally begin work earlier than hunter-gatherer children (*172*). Among Agta forager-farmers of the Philippines, time allocation to farming is negatively associated with sedentary leisure time (*173*). For Tsimane, greater time allocation to horticulture earlier in life positively predicts ultrasound-derived proxies of radial stiffness (*153*). Horticulture is routinely performed by both sexes and involves use of machetes that are swung while leading with the elbow and alternating arms when fatigued. Bioarchaeological studies show an increase in upper limb strength corresponding to greater nutritional reliance on native seed crops from ~50 BC to 1050 AD, particularly for women, who may have been primarily responsible for processing these crops (*18*, *174*, *175*). Generally speaking, mechanical strains in the upper limbs from routine tasks (e.g., carrying infants or either fallen tree trunks, firewood, or water and grinding, mashing, pounding, and scraping plant- or animal-based foods) have not been as thoroughly studied as lower limb strains amidst adaptations to bipedality. Study of contemporary subsistence populations can help illuminate the impacts of routine tasks on diverse MSK system components, even if our ability to make inferences about the past based on insights from the present is limited.

It is now clear from genomic, bioarchaeological, and paleoclimatic studies that during the process of plant and animal domestication, exposome changes did not occur simultaneously, uniformly, or unidirectionally. Domestication was instead a complex process unfolding in a regionally heterogeneous manner. In the New World, plant domestication occurred thousands of years before animal domestication, whereas the opposite was true in Africa, Arabia, and India (*176*). In some regions, transition from foraging to farming occurred over several millennia, whereas in others, it occurred more rapidly. To the extent that contemporary subsistence populations provide an accurate window into lifeways of past populations, early Holocene farmers likely pursued a mixed economic strategy including foraging [cf. (*177*)]. Foragers likewise extensively managed wild plants before agriculture (*176*). Simple binary contrasts between foragers and farmers can therefore be misleading (*22*), and causally linking precise exposome changes to MSK health outcomes since the early Holocene is challenging. For example, in the Nile valley, relative to their foraging predecessors living ~11 to 15 kya, early farmers from ~6 to 7 kya exhibit poorer health (proxied by greater prevalence of linear enamel hypoplasia and reduced stature), but later farmers from ~4 to 6 kya exhibit similar or improved health (*163*, *178*). In other regions, initial transition to agriculture is associated with increased stature, followed by longer-term stature declines or either no temporal change in stature or subtle patterns varying by sex (*22*). Whether sex differences reflect sex-specific costs of reproduction or interactions between reproductive effort and other exposome features is an open question. In central and northern Europe, stature increases from ~4-8 kya roughly coincide with the timing of selective sweeps for lactase persistence. This suggests that a shift toward consumption of dairy products mitigated stressful exposures that may have been associated with early farming including nutritional stress from reduced dietary diversity or pathogen stress. In other regions, declines in stature and lower limb bone strength precede the emergence of agriculture (*179*–*182*), and sometimes no differences in limb robusticity are found between agriculturalists and pre-agriculturalists [e.g., (*183*)]. An expanded framework incorporating diverse exposome features can improve our understanding of human biological transformations associated with socioeconomic, epidemiological, and demographic transitions.

In this Review, insights from human behavioral ecology, paleoanthropology, primatology, functional morphology, evolutionary medicine, and epidemiology have been used. We propose that transition to bipedalism beginning ~6 to 8 Mya can be conceptualized as an early mismatch scenario, increasing hominin susceptibility to certain chronic MSK conditions due to novel mechanical loading environments. Anatomical regions (e.g., the thoracolumbar spine) that were not previously exposed to significant recurring mechanical forces from an increasingly bipedal and terrestrial extractive foraging niche would be susceptible to structural deterioration. Anatomical regions (e.g., the shoulder) that were once regularly exposed to significant forces from a largely quadrupedal and semi-arboreal lifestyle, but which experienced major changes in loading regimes accompanying locomotor transition, could also be susceptible to deterioration. Examples of chronic MSK conditions that early hominins may have commonly experienced include facet joint OA, kyphosis, degenerative disc disease, fatigue fracture-induced spondylolysis, fragility fractures, and conditions which may not be directly discernible from the fossil record (e.g., chronic MSK pain). Certain MSK conditions including poor posture need not simply result from the extensive longevity observed in modern humans. They can be prevalent among children and young adults and result from high physical activity levels (*184*) that likely characterized past populations.

We propose that an interaction between phylogeny and ecology places humans at uniquely high risk of chronic MSK conditions. Given the suite of ancestral and derived morphological traits that humans have, we experience unique mechanical stresses from orthograde posture, bipedal locomotion, and distinctive physical activity patterns (e.g., expanded day ranges; carrying objects varying in mass sometimes for long distances) used to harvest energy-rich nutrients and care for offspring. Under certain conditions, physical activity–based mechanical loading can strengthen MSK tissues and minimize their risk of deterioration with aging. But early-life exposure to habitually high activity levels can also promote tissue damage accumulation from wear-and-tear, increase risk of traumatic injury and disability which compromise productivity, and reduce investments in somatic maintenance given constrained energy expenditure. Constraints result in part from evolutionary life history trade-offs favoring energetic investments in pathogen defenses and higher fertility. Relatively recent exposome changes (e.g., promoting obesity within the last few decades) can either exacerbate effects of MSK traits that were already mismatched earlier in hominin evolutionary history or yield distinct mismatches that further increase the burden of human MSK conditions.

According to this perspective, certain chronic MSK conditions should be evident in early hominins [e.g., (*25*, *26*, *28*)] but nonexistent or relatively rare and less severe in Miocene and Pliocene primates committed to quadrupedal locomotion. These hominin conditions should long predate other now common noncommunicable diseases (e.g., coronary artery disease) which are often hypothesized to result from interactions between evolved metabolic phenotypes and relatively recent exposome changes promoting chronic positive energy balance [e.g., (*2*)]. Nowadays, several noncommunicable diseases affecting cardiometabolic and other organ systems aside from the MSK system reliably cooccur with various chronic MSK diseases and share upstream risk factors that were rare or absent in the past (e.g., physical inactivity and obesity). But health and aging profiles among contemporary small-scale subsistence populations reveal that certain chronic diseases need not cooccur. The fact that high reproductive effort depletes maternal skeletal mineral reserves [Figs. 3 and 4; (*112*)], increasing older women’s fragility fracture risk (*70*) but that older women’s cardiovascular and cognitive functioning can also remain relatively well-preserved (*1*, *2*) indicates that trade-offs between reproduction and somatic maintenance depend on proximate physiological mechanisms (*104*) and that organ systems within individuals senesce at varying rates. Future research into biological aging profiles among contemporary subsistence populations offers promise to reveal mechanisms that may underlie genetic variants with antagonistic pleiotropic effects [cf. (*185*, *186*)].

This is not the first time that it has been proposed that certain chronic human MSK conditions are mismatch diseases [e.g., (*4*, *15*, *187*)]. Nor is it the first time that it has been proposed that changes in mechanical loading regimes associated with transition to orthograde posture and bipedal locomotion resulted in certain chronic MSK conditions that are unique to humans [e.g., (*24*, *41*, *45*)]. Here, insights from this prior work are synthesized, and recent evidence linking human MSK functioning to four specific exposome features that are common to small-scale subsistence populations is incorporated. The value of considering exposome features both individually and in interaction with physical activity–based mechanical loading is further highlighted.

Regarding other research priorities in extant populations, characterizing movement ranges and MSK tissue responses to loading in humans and other great apes under naturalistic conditions using noninvasive methods [e.g., surface electromyography; cf. (*188*)] will help clarify what a mismatch actually entails. Under what conditions and to what extent are MSK system components such as joints and bone and other tissues overloaded, underused, and misused during performance of routine economic and parenting tasks? Are degenerative MSK conditions more likely in anatomical regions that are excessively or insufficiently loaded (*41*, *45*)? What types of movements and mechanical loading strengthen tissues during development and help maintain their structural resistance with aging considering evolutionary life history trade-offs and phylogenetic constraints on energy expenditure? To what extent do more recent exposome changes (e.g., those promoting chronic positive energy balance and obesity) either exacerbate effects of MSK traits that were already mismatched earlier in hominin evolutionary history or yield distinct mismatches?

To conclude, in vivo study of the human MSK system in small-scale subsistence populations provides an opportunity to discover effects of exposome features that may be invisible in the fossil or genomic record and in studies of higher-income populations but are nevertheless relevant for understanding variation in health and aging. Here, we focused on MSK system responses to (i) high physical activity–based mechanical loading from subsistence effort, (ii) high pathogen burden, (iii) variable nutrient intake including potential for limited micronutrient supply, and (iv) relatively early and rapid reproduction characteristic of natural fertility regimes. Human MSK biology and health have been profoundly influenced by exposome alterations, many of which were endogenous. Integrated biobehavioral studies over the life course in subsistence populations may help reconcile mixed findings in past populations, uncover overlooked risk factors for MSK conditions in present populations, and identify factors promoting healthy development and aging.
